# Patient and surgeon comfort in vitreoretinal surgery performed with Sub-Tenon’s Anaesthesia

**DOI:** 10.22336/rjo.2021.28

**Published:** 2021

**Authors:** Fabrizio Franco, Lidia Vicchio, Giuseppe Ruben Barbera, Gianni Virgili, Fabrizio Giansanti

**Affiliations:** * University of Florence, Department NEUROFARBA, Eye Clinic, Florence, Italy

**Keywords:** Sub-Tenon’s anaesthesia, vitreoretinal surgery, local anaesthesia, alternative anaesthesia

## Abstract

**Background**.

Since Stevens first introduced Sub-Tenon’s anaesthesia into cataract surgery it has shown itself to be a safe, simple, and efficient technique. The advantages of this type of block are comparable to those of sharp needle anaesthesia and complications are minimal. Several studies have found that the anaesthesia provided by Sub-Tenon’s capsule injection is as good as or better for cataract surgery than that achieved by retrobulbar injection, but the efficacy of Sub-Tenon’s block in vitreoretinal surgery is less well established.

**Methods**.

We performed 50 vitreoretinal procedures; 50 eyes received a Sub-Tenon’s injection of a 5 ml mixture (50:50) of lidocaine and ropivacaine, plus 15 IU mL-1 of Hyaluronidase.

**Results**.

In 45 cases, only one injection was needed to achieve sufficient anaesthesia and akinesia; in 5 cases a second injection was performed five minutes after the first. Mean surgical time was 45.7 minutes. After surgery, each patient was asked to indicate his value on the VAS pain scale. Mean VAS degree was 2.4. In 7 cases, VAS was > 3 and the pain was successfully managed with the administration of paracetamol in the postoperative period. No light perception was detected at the end of surgery in 33 patients. All cases with 2 injections had no light perception at the end of surgery. Anaesthesia lasted throughout the surgery in all cases. The surgeon performed all surgery comfortably and with no difficulty.

**Conclusions**.

According to our experience and to a growing body of evidence, Sub-Tenon’s anaesthesia appears to be a safe, simple, versatile, and effective technique and should be considered as a real alternative method of anaesthesia in vitreoretinal surgery.

## Introduction

Sub-Tenon’s anaesthesia, pinpoint block, or episcleral block, was described for the first time in 1884 by Turnbull [**1**] and subsequently by Swan [**2**] in 1956, but it is only in the 1990s that Stevens [**3**] introduced the technique in cataract surgery. Since then, this technique has proven to be a safe, well-tolerated, effective, and versatile alternative to peribulbar, retrobulbar, or topical anaesthesia in anterior and posterior segment eye surgery [**4**-**7**]. Sub-Tenon’s anaesthesia allows physicians to achieve good analgesia and akinesia, avoiding the risks associated with needle techniques, such as globe perforation, retrobulbar haemorrhage, optic nerve damage, and inadvertent injection into the subarachnoid space [**8**-**11**]. So far, Sub-Tenon’s injection has not only been used increasingly for cataract surgery but also for vitreoretinal surgery, strabismus surgery, pan-retinal photocoagulation, optic nerve sheath fenestration, retinopathy of prematurity, glaucoma, uveitis, capillary haemangioma, chronic pain management, and for the therapeutic delivery of drugs [**12**-**16**].

Tenon’s capsule is a fascial layer of connective tissue that surrounds the globe and invests the extraocular muscles. Anteriorly, the fascial sheath continues in the conjunctiva 1.5 mm posterior to the limbal margin. Posteriorly, the layer fuses with the meninges near the point where the optic nerve enters the globe. The potential space that exists between the inner surface of the capsule and the outer sclera is known as the Sub-Tenon’s or epis¬cleral space. This space contains numerous delicate bands of connective tissue and it is filled with lymphatic fluid that provides a low-resistance environment for globe excursion, and it continues into the subdural space. The capsule’s innervation is given by the short ciliary nerves that penetrate its posterior por¬tion on their way to the globe. The tendons of all the 6 extrinsic eye muscles penetrate Tenon’s capsule posteriorly to the equator of the globe. Probably, akinesia is achieved by blocking the motor nerves as they pass through the space prior to innervating the extraocular muscles [**17**].

Three main drugs are used for regional eye blocks: bupivacaine, lidocaine, and ropivacaine. Bupivacaine has been reported to be about four times more potent than lidocaine and is similar to ropivacaine [**18**,**19**]. Lidocaine penetrates very easily into tissue, and as a result, it is the fastest acting but at the same time, it has the shortest duration of action. In addition, some recent studies have proposed the use of ropivacaine for ocular blocks due to its duration of action and evidence of fewer cardiovascular effects compared with bupivacaine [**20**-**22**]. 

Some adjuvant agents can be added to the anaesthetic solution injected. Vasoconstrictors (e.g., epinephrine) are applied to increase the intensity and duration of the block, minimizing bleeding and systemic absorption. Nevertheless, the role of Epinephrine is questioned because of possible complications, such as vasoconstriction of ophthalmic arteries and increased risk in older patients with cardiovascular and cerebrovascular disease [**23**]. Another substance that can be added to anaesthetic solutions is Hyaluronidase, which catalyzes the hydrolysis of hyaluronan, a constituent of the extracellular matrix (ECM), increasing tissue permeability. Hyaluronidase appears to improve the effectiveness and quality of the block with very rare side-effects [**23**-**25**]. Furthermore, other studies have been conducted to determine whether pH alterations could offer significant benefit in Sub-Tenon’s block (STB). In fact, alkalinization of local anaesthetic agents has been shown to prolong the duration of needle blocks, but no differences have been found when modifying pH in Sub-Tenon’s Block [**26**].

## Materials and methods 

We enrolled 50 eyes of 50 patients, 27 right eyes and 23 left eyes, between September 2019 and March 2020. Patients underwent vitreoretinal surgery for macular disease (28 males and 22 females). Mean age was 73.3 years. We performed vitrectomy on 5 retinal detachments, 6 vitreous haemorrhages, 4 vitreomacular tractions, 14 macular holes (8 of them combined with cataract surgery) and 21 macular puckers (15 of them combined with cataract surgery). In all cases, we used Alcon vitrectomy system Constellation® (Alcon, TX, US): 33 procedures were performed with 25g 10,000 cuts and 17 procedures with 23g 10,000 cuts. In all cases, we performed Sub-Tenon’s anaesthesia with a 5 ml mixture (50:50) of lidocaine and ropivacaine, plus 15 IU mL-1 Hyaluronidase. All surgical interventions were performed by the same surgeon (F.F.). A complete preoperative evaluation for vitreoretinal surgery was performed (slit lamp examination for anterior and posterior segment, tonometry, Best Corrected Visual Acuity, Optical Coherence Tomography). All surgery was performed under Sub-Tenon’s block (STB). Anaesthesia was performed directly on the operating table. 

After disinfection of the eyelid skin with povidone iodine 10% and application of ophthalmic sterile cloth, the Lieberman eye Speculum was positioned, and anaesthetic eye drops (lidocaine 4%) were instilled on the ocular surface. To complete disinfection, povidone-iodine 5% was instilled in the conjunctival fornix for 3 minutes and washed out with Balanced Saline Solution. A small incision was made in the conjunctiva of the inferonasal sector, 5 mm from the limbus, with blunt scissors, and the tenon capsule was dissected until the sclera was released. A dedicated blunt needle (Charleux Cannula) was inserted under the conjunctiva and tenon and it was glided over the surface of the eye. 

The injection started when the equator was reached. The anaesthetic solution was composed of: 5 ml mixture (50:50) of lidocaine 4%, ropivacaine 0,5% and 15 IU mL-1 Hyaluronidase. In 90% of cases, a single injection was sufficient to obtain anaesthesia and akinesia after only 3 minutes. In 5 cases, after 3 minutes, anaesthesia but not akinesia was obtained after the first injection, therefore more anaesthetic was needed in the form of another Sub-Tenon’s block. During surgery, the comfort of surgeon and patient was evaluated. Regarding the surgeon’s comfort, the time to reach complete anaesthesia and akinesia, cleanliness of the surgical field, difficulties in surgical maneuver (trocar insertion, indentation, peeling, suturing of sclerotomies), were estimated. 

For the patients’ comfort, the following elements were assessed: pain during aforementioned-phases of surgery and comfort with absence of visual stimuli (no light perception during surgery).

After surgery, patients returned to the wards. Two hours after the end of surgery they were asked to indicate the corresponding degree on a Visual Analog Scale for pain (VAS Pain). The VAS pain scale is self-completed by the respondent: he is asked to place a line perpendicular to the VAS line at the point that represents his pain intensity. 

## Results

In 45 cases, only 1 injection was needed, in 5 cases 2 injections, the mean number of injections being 1.1. The second injection was performed 5 minutes after the first to achieve better akinesia. A residual activity of the lateral rectus can typically exist after the first Sub-Tenon’s injection. Finally, we reported total akinesia in all cases. The mean surgical time was 45.7 minutes. In all cases, the surgical maneuvers were performed without difficulty. Trocar positioning did not cause pain, no discomfort on the ocular surface being reported during surgery. The sclerotomies were sutured in all patients who underwent 23G vitrectomy (17 patients – 34%), 3 of them reported mild pain during the procedure. The surgeon did not find any difficulty during the surgical procedure, specifically during the peeling or indentation for peripheral vitrectomy. The mean VAS degree was 2.4. Three cases had a VAS score of 7 and needed the administration of paracetamol 1 gr IV in the postoperative period. In 4 cases, the VAS score was 6 and these patients required oral paracetamol after surgery. Nine cases had a VAS score of 3, 14 cases had a VAS score of 2 and 20 cases had a VAS score of 1. In these cases, no analgesic drug was required in the postoperative period. No light perception was detected at the end of surgery in 33 patients, including all the cases who received 2 injections. The incidence of no-light perception was not correlated to the preoperative condition, age, and gender. Anaesthesia lasted throughout the surgery in all cases. We reported only minor complications: 29 patients suffered from subconjunctival haemorrhage. The surgeon performed all surgical interventions comfortably and without difficulty.

## Discussion

Complications associated with STB are mainly minor although rare major complications have been reported. The most frequent are: pain on injection site (44%), chemosis (25%–60%), subconjunctival haemorrhage (20-100%), and over-spillage of LA during administration. The incidence depends on the choice of cannula used, volume of LA and dissection technique [**27**-**29**]. Significant sight- and life-threatening complications of STB are rare but have been reported. These include short-lived muscle paresis and orbital and retrobulbar haemorrhage (<1%). Other complications are related to muscular trauma, optic neuropathy, retinal and choroidal vascular occlusion, and even cardiorespiratory collapse and death [**30**-**34**]. 

Nevertheless, Retrobulbar block is related to more frequent major complications [**35**]; an analysis of 26,045 Sub-Tenon’s and 11,134 Needle Blocks, showed only 4 cases of retrobulbar haemorrhage in the former vs. 8 cases in the latter [**36**].

Moreover, the risk of perforation is increased with retrobulbar block, leading to vitreous haemorrhage, retinal holes (especially at the posterior pole) and retinal detachment [**37**,**38**]. Intra-vitreous injection of anaesthetic may cause a raise of IOP with occlusion of the central retinal arteria or ischemic optic nerve neuropathy [**38**]. 

STB is not related to a risk of intravascular injection, which is a possible complication of retrobulbar or peribulbar anaesthesia, with life-threatening effects; nor is it related to reabsorption through the meninges that envelope the optic nerve before entering the scleral globe [**38**]. 

In our clinic (Eye Clinic, AOU Careggi, Florence, Italy) the Retro-bulbar block is performed under sedation with Propofol administration, increasing patients’ risks and side effects (significant decrease in respiratory rate, respiratory depression, apnea, low blood pressure, bradycardia).

Moreover, an observation time of 2 hours after surgery is mandatory because of this administration of systemic anaesthesia (even if it is of short duration) and the risk of local anaesthetic reabsorption.

STB is simpler to perform compared to retrobulbar block, with a shorter learning curve. Increased difficulty performing Sub-Tenon’s block should be considered when: STB has already been administered in the same quadrant; a patient underwent previous strabismus surgery or scleral buckling; infection in the orbit and eye trauma. Moreover, there is evidence that needle blocks should be avoided in patients who are treated with anticoagulants and non-steroidal anti-inflammatory drugs and STB should be preferred; the same applies to patients with long globes [**39**]. A shorter time is necessary to obtain sufficient anaesthesia and akinesia with STB compared to sharp needle block, allowing less downtime in surgery. 

In our study, we have reported that STB is very efficient in providing akinesia with 1.1 mean number of injections and controls pain with a mean VAS degree of 2.4. In 6 cases, the VAS was > 3 and pain was successfully managed with the administration of paracetamol in the postoperative period. Furthermore, 66% of patients experienced no light perception during surgery, preventing uncomfortable visual sensations during the procedure. All surgical interventions were conducted comfortably for both the surgeon and the patients. Anaesthesia lasted throughout the surgery in all cases (only 3 patients reported mild pain during suturing) and the only complication reported was subconjunctival haemorrhages in 29 patients. With STB anaesthesia, the mean time of post-operative observation was shorter than with peribulbar retrobulbar injection. 

The temporary absence of light perception was a transient effect caused by the anaesthetic drugs and did not impair visual function.

Several mechanisms have been analyzed to explain light perception loss after Sub-Tenon’s block: the blocking of conduction through the optic nerve caused by the anaesthetic or low perfusion of the optic nerve and retina caused by high intra-orbital pressure or intraocular pressure. Retinal ischemia is not very probable: generally, retinal ischemia causes irreversible damage after a few minutes but in our study and in literature all patients had total recovery of visual acuity [**40**]. 

On the other hand, local anaesthetics can prolong the refractory period by inhibiting the passage of Na+ into the nerve cell membrane and reducing temporarily the excitability and conductivity of the neurons [**41**]. 

Sharp needle techniques are still the most common in posterior segment surgery worldwide. We have proposed an alternative using STB, which resulted in fewer risks, faster execution, and equal efficacy. Although a prospective randomized controlled trial to directly address a comparison of the safety profile of the various, local anaesthetic techniques has never been carried out, there is growing evidence that STB is as effective as retrobulbar injection in providing local anaesthesia in vitreoretinal surgery [**42**]. 

## Conclusion

At present, there is no orbital regional block technique that is 100% well tolerated. However, good evidence appears to support Sub-Tenon’s block as the safer option. It offers excellent surgical results, with important complications being uncommon. Regarding vitreoretinal surgery, the results of our study, and a growing body of evidence, demonstrated that STB is a simple, effective, and good alternative to the current standards of practice. While waiting for further prospective, randomized, double-blinded studies we would encourage the extension of this technique, the application of which is still modest in posterior segment surgery.

**WHAT IS KNOWN**

Sub-Tenon’s anaesthesia is a safe, effective, and simple procedure. The application of Sub-Tenon’s Anaesthesia should be prompted in vitreoretinal surgery. 

**WHAT IS NEW**

Sub-Tenon’s anaesthesia can provide a good analgesic effect after surgery. Surgical maneuvers are not harder under Sub-Tenon’s anaesthesia. 

**Conflict of Interest Statement**

The authors declare no competing interests. 

**Informed Consent and Human and Animal Rights statement**

Informed consent has been obtained from all individuals included in this study.

**Authorization for the use of human subjects**

Ethical approval: The research related to human use complies with all the relevant national regulations, institutional policies, is in accordance with the tenets of the Helsinki Declaration, and has been approved by the review board of the University of Florence, Department NEUROFARBA, Eye Clinic, Florence, Italy.

**Acknowledgements**

All authors contributed equally to the manuscript and read and approved the final version of the manuscript.

**Sources of Funding**

Support was provided solely from institutional and departmental sources (University of Florence, Department NEUROFARBA, Eye Clinic, Florence, Italy).

**Disclosures**

None.
